# Mediating Trauma and Anxiety: Letters to Françoise Dolto, 1976-1978

**DOI:** 10.1007/s10912-020-09625-7

**Published:** 2020-04-16

**Authors:** Richard Bates

**Affiliations:** Department of History, University of Nottingham, Nottingham, UK

**Keywords:** Radio, Psychoanalysis, Letters, France, Children, Correspondence, Trauma, Child-rearing, Parenting, Psychotherapy

## Abstract

Françoise Dolto (1908-88) was a prominent French cultural figure thanks to her practice of dispensing psychoanalytically-informed child-rearing advice via the radio. From 1976 to 1978, on her show *Lorsque l’enfant paraît*, she responded to thousands of letters sent in by listeners requesting help with parenting problems and personal questions of a psychological nature. The article explores Dolto’s cultural position as a child psychoanalyst – understood in the 1960s and 1970s as a radical profession – but from a conservative, Catholic background. It then examines a sample of the letters sent in to her show, analysing the demographics of the correspondents and highlighting their most common concerns. Finally the article studies the relationship between psychoanalysis and trauma. It indicates the anxiety that a psychoanalytic understanding can cause, by reinforcing parents’ fears that childhood traumas are common, can be created unwittingly by parents, and lead to major psychological consequences in later life.

In 1968-9, the French psychoanalyst Françoise Dolto hosted a radio phone-in show on the commercial radio station Europe 1 entitled *SOS Psychanalyste!* The exclamation mark revealed the ambiguity of the show’s intent. Listeners were offered the opportunity to discuss an ‘emergency’ personal problem with a qualified professional, but the show was also a form of entertainment. Dolto’s participation – soon identified despite hiding her identity behind the label ‘Docteur X’ – attracted criticism from the French psychoanalytic and psychotherapeutic community. How could one claim to dispense psychoanalysis, a treatment requiring a long-term commitment to understanding the deepest workings of the unconscious mind, in five minutes to a disembodied stranger? The concept was seen as bringing psychotherapeutic medicine into disrepute; Dolto was threatened with suspension from the Ordre des Médecins (Sauverzac 1995, 365). The show was cancelled after one season.

However, Dolto did not permanently renounce the idea of finding ways to bring psychoanalysis to a popular audience. Born in 1908 to a wealthy Catholic family in Paris’ prim 16th arrondissement, in which she experienced a cloistered childhood not dissimilar to that described by Simone de Beauvoir in *Memoirs of a Dutiful Daughter*, she had numerous connections to the French media and political establishment before 1960 (Grellet and Kruse 2004; Dolto 1986; Dolto 1989). Her brother, Jacques Marette, had been Minister for the Post and Telecommunications under Charles de Gaulle, while her son, Carlos, a friend and sometime driver to Johnny Halliday, had won fame as a radio entertainer and pop star. Popular engagement had been a theme of Dolto’s career since the Second World War. As early as 1946, she had a column in the popular magazine, *Femmes françaises*. She wrote for various psychological and Catholic publications, such as *Psyché*, run by the Catholic writer Maryse Choisy; *Études carmélitaines*, founded in 1949 by a liberal-minded priest; and the journal of the *École des parents*, an association – also of Catholic inspiration – with the aim of diffusing practical parenting knowledge and methods. She occasionally spoke on the radio in the guise of a child psychology expert, for example in 1950 to argue in favour of sex education. She took advantage of her brother’s ministerial position to commence what became an annual tradition in France, ‘la réponse du père noël’ – a friendly, generic reply, sent at state expense, to children’s Christmas lists.

In the years following 1968, France was undergoing a series of significant cultural changes, often collectively referred to as *les années ‘68* in reference to the role of the student and worker demonstrations of May 1968 as a trigger for the transformations in question (Dreyfus-Armand et al. 2008). These changes included the growth of feminist, homosexual, and immigrant consciousness; a shift in the place of youth in society; and changes in educational philosophy. In addition, the 1970s were the culmination of a longer period of economic and social transformation that had begun after the Second World War. French economic growth in the 1960s was the fastest in Europe and second worldwide only to Japan. Cities and suburbs grew rapidly through internal and external immigration, life expectancy increased by some 8-10 years, and a new consumer and leisure-oriented society took shape – indicated by the growth in TV ownership from 9% of households in 1958 to 80% in 1974 (Cholvy and Hilaire 2002, 164-5).

Above all, May 1968 embodied the weakening or discrediting of various forms of authority: that of De Gaulle and his government; the authority of the police, tainted by association with colonial and Vichy repression; the authority of the trade union Left, perceived as too embedded in the system and as having vacated the terrain of resistance and radicalism; and that of Marxist political analysis, discredited by the repression of the Prague Spring rather than events in Paris. For historian Rod Kedward, this period witnessed ‘final disintegration of the archaic ideological opposition to social change which had limped on in the ranks of the political right throughout the 1950s and had sheltered behind the authoritarian rule and nationalism of de Gaulle in the 1960s’ (Kedward 2006, 368). A further authority that declined significantly in *les années ‘68* was that of religion. Regular church attendance dropped precipitately, from 30% of the population in 1961 to 14% in 1975, with most of the decline happening after 1969 (Hilaire 2000, 255). The number of ordinations into the clergy fell sharply.

Following the 1968 events there was thus a strong appetite for new ideas and radical theories. Maoism was one of these, attracting intellectuals such as the philosopher Alain Badiou and the writer Philippe Sollers. Psychoanalysis was another. As a discipline it had had a relatively slow start in France; the Société Psychanalytique de Paris, the institution set up in 1926 to organise and promote it, had only 24 full members in 1939 (Roudinesco 1990). However after the Second World War it had prospered and established a significant foothold within the state medical system, such that by 1967, young medical graduates entering the psychiatric service felt under pressure to undertake a personal analysis (Cottraux 2005, 252). The movement was however fragmenting, with splits in 1953 and 1964 over how far psychoanalysis should be treated as a purely medical as opposed to a philosophical discipline, whether it should be practised by people without a medical degree, and increasingly, over the authority of Jacques Lacan. Lacan’s seminars, delivered at the École Normale Supérieure (at the invitation of Marxist philosopher Louis Althusser) from 1964, drew a wide audience from well beyond medicine and psychiatry, and appeared to announce the arrival of psychoanalysis as a central discipline in French intellectual culture. His theoretical radicalism and disregard for the norms of psychiatric and psychoanalytic practice (such as his use of variable-length, often very short, therapeutic sessions as opposed to the standard therapeutic ‘hour’) chimed with the predominant mood of questioning of authority.

Public interest in psychoanalysis spiked after 1968, and publishers soon sought to capitalise. Lacan’s *Écrits*, first published in 1966, were re-printed in a popular two-volume edition in 1969. A number of younger psychoanalysts, such as André Green, Didier Anzieu, and Daniel Sibony, published their first books in this period, works which were not orthodox medical studies but instead pushed psychoanalysis into new areas of cultural and literary criticism. Another sign of the times was the creation of a Department of Psychoanalysis at the University of Paris VIII, under the control of the Lacanian disciple Serge Leclaire. By 1972, the increased cultural presence of Freudian thinking was producing its own radical backlash, for example in Gilles Deleuze and Félix Guattari’s *Anti-Oedipus*, and among some feminist campaigners.

Françoise Dolto – who remained aligned with Lacan throughout the splits in the psychoanalytic movement – was a direct beneficiary of the growing prominence of psychoanalysis. Despite her regular writings in journals and magazines, she had not published a book-length work since her doctoral thesis, *Psychoanalysis and Paediatrics*, completed in 1939. In 1971 she was approached by an editor at the prestigious Éditions du Seuil who offered to re-publish that work, plus a book-length psychoanalytic case-study, *Le cas Dominique*. Both were reprinted as mass-market paperbacks a few years later, achieving solid sales and positive press coverage. And in 1976, a producer at the state broadcaster, Radio France, invited her to make another attempt at an audience-participation radio show.

Dolto was an attractive choice for Radio France not only because she was a respected psychoanalyst with experience of broadcasting, but also because her position with regard to the radical legacy of May ‘68 was ambivalent. In effect, if many people were looking for change and novelty after 1968, there were also large groups of French people who felt disconcerted by the sense of a world being uprooted, and seeking reassurance and continuity. The massive pro-Gaullist demonstration of 30 May, eclipsing the mobilisations of the students and workers, and the large majority won by the centre-right in the elections of June 1968, were one manifestation of this. More broadly, the decline of France’s traditional rural economy and its attendant behaviours and attitudes (such as religious observance), and the growth of a technocratic, bureaucratic society in its place, made it seem to some as if a whole way of life was disappearing. This gave rise to nostalgia for a lost or fading artisanal world associated with authenticity and freedom from bureaucratic restraint, expressed in literature and popular culture and in the writings of thinkers such as the Jesuit intellectual Michel de Certeau.

As a Christian who was old enough to remember both World Wars, who had grown up under the influence of conservative and even far-right thinking in the 1920s and 1930s (she had been a firm supporter of Marshal Pétain in 1940 and worked for a pro-Vichy think tank during the Occupation) Dolto was well placed to appeal also to this second current. Her claim to expert authority was based in large part on her length of experience: she had qualified as a doctor and psychoanalyst in 1939 and had been a paediatric consultant at a Parisian hospital since 1940. She freely admitted that her advice drew on grandmotherly wisdom as often as it did on Freudian theory. She did not identify with feminism, and defended conservative positions on subjects such as abortion and gay rights. Her work tended to stress the importance of the classic nuclear family and the active presence of two heterosexual parents, which drew her a degree of criticism from feminists and homosexual rights activists, including for example Luce Irigaray (Irigaray 1985, 63).

Dolto in 1976 was thus in the unusual position of being able to speak to both aspects of the cultural climate of the time. She gained a certain progressive cachet from her association with psychoanalysis. She was becoming known as a liberal voice on some issues, notably sex education and educational reform, where she argued for school environments designed to foster children’s autonomy and creativity and criticized the obedience and rigidity that she argued had historically characterized France’s state education system. Yet in other ways she appeared to be out of step with the radical spirit of *les années ‘68* in ways that would reassure a more conservative audience.

It would appear to have been this unusual balance which encouraged Radio France to hire her. Mindful of the failure of *SOS Psychanalyste!*, Dolto was initially reluctant to make another venture into radio, but eventually agreed. This time, however, there would be a crucial difference – instead of phone calls, listeners would send in their psychological dilemmas via letter, in response to broad themes suggested by Dolto.

The new programme would be called *Lorsque l’enfant paraît* (‘When the child appears’). It would focus on helping parents understanding the psychology of their children and dealing with problems with a psychological component – bed-wetting being a frequent example. It went out on weekdays at 3 p.m. and each episode lasted just fifteen minutes. Despite this apparently unpromising slot, the programme was an enormous success. It made Dolto into a household name in France, leading to three spin-off books that sold hundreds of thousands of copies (Dolto 1977-79). Transcripts from the earlier show *S.O.S. Psychanalyste!* were also published (Dolto 1976). By 1978 Dolto felt that she could no longer take on new clients to her psychoanalytic practice, because her degree of fame was such that she could no longer be a neutral presence.

The archive of letters sent to the programme was preserved initially by Radio France and subsequently by the Archives Françoise Dolto, which were in turn transferred to the Archives Nationales in 2015. It totals around 6,000 letters, offering an insight into the psychological concerns and anxieties of a cross-section of French society of the 1970s. For the purposes of this article, I examined a randomly-selected sample of 266 of these letters.

To begin with, the show received modest volume of correspondence – around 40-60 letters per week for the first two months. Early letters mainly came from mothers and were concerned with direct questions of child-rearing, such as toilet training, sibling rivalry, breastfeeding, or how to tackle subjects such as sex or death with children. As the show progressed, the number of letters received rose to between 100 and 150 per week, and the average length of the letters also increased. Letters began to come in from adults and adolescents regarding their own psychological problems (as opposed to those of their children), and doctors, teachers and nurses wrote in offering their own perspectives. Dolto received numerous requests for book suggestions, for the names and addresses of recommended psychotherapists (or requests to be taken into therapy with Dolto directly, all of which were refused) or invitations to participate in lectures, conferences, governmental consultations, and various other events.

Around three-quarters of the letter-writers were female. This gender imbalance disguises an even greater one when it came to parenting, for many of the letters from men were written in a professional capacity (as doctors, teachers, members of associations, or employees of state agencies) or were penned by general listeners without a direct personal issue to raise. Of the 266 letters in the sample, 107 were written by mothers raising issues regarding their children, compared to only nine from fathers (despite repeated requests from Dolto for more fathers to write in) and five from both parents jointly. Nine letters came from grandmothers, and only one from a grandfather. A further six were from pregnant women.

Letters came in from all parts of France and sometimes beyond – around 7% of the letters were sent from abroad, mostly from Germany, Belgium and Switzerland. The number of letters received from each French region was mostly in proportion to its population, but with some exceptions. Letters were disproportionately likely to come from wealthier regions such as those around Paris and Lyon, and the Pays de la Loire, Midi-Pyrénées and Auvergne. Disproportionately few letters were received from poorer regions such as Lorraine, Burgundy, Picardy and Languedoc. This pattern was repeated in the letters from within Paris. Most Parisian correspondents lived in the wealthy districts of western Paris, not the working-class areas of the north and east. This may simply demonstrate the truism that wealthier people have more time and inclination to write letters in to radio shows, but it may also say something about the social conservatism of Dolto’s core audience. For the most part, the letters are carefully written, without major errors of spelling or grammar, and most writers appear as to have, at least, a reasonable level of secondary education.

The subject of trauma and associated lexical fields are a recurring theme in the correspondence. It is striking, however, that in most cases the trauma in question is one that is feared or hypothetical rather than one that has definitely occurred. Mothers write of their fear that a particular event or set of circumstances will traumatise their child or leave behind negative ‘traces’ or ‘consequences’ for their child, or to ask Dolto’s opinion as to whether or not a certain experience could be traumatic for a child. The very first letter received by the show, in September 1976, asked whether children who are obliged to repeat a year of schooling would be forever scarred (*sortent marqués à jamais*) by the experience.^1^ Other representative examples include a letter from a 23-year old secretary in Chantilly, who wrote to Dolto of her difficulty in dealing with the crying of her 8-month old baby. Sometimes it all became too much and she resorted to giving the baby a smack on the bottom (*fessée*) to silence him. She asked Dolto to tell her ‘if this will have traumatised him and whether it will have psychological after-effects (*des séquelles*)’? (September 1977, 7:3:24). One mother wrote of her concern about children being traumatised by finding out about the realities of human sexuality too early, or in the wrong way; another asked whether allowing children as old as five or six to sleep in the same room as their parents would have long-term psychological repercussions (October 1976, 1:2:15; 1:1:10). A further correspondent worried about the negative consequences for her child of a difficult weaning (September 1977, 7:3:2). One listener even wondered about the possibility of pre-natal trauma, wanting to know ‘how the psychical and nervous balance of a pregnant woman can be experienced by the child she is carrying’ (April 1978, 13:34:90). The language of error accompanies that of trauma; the mothers who wrote to Dolto were frequently afraid of making mistakes. ‘I’m afraid that it’s too late, I’m realising that I’ve committed major errors,’ wrote one mother of three children, aged between nine and twelve (April 1977, 5:29).

As the show’s popularity increased and Dolto’s reputation spread, two other types of letter emerge from the archive. First, some writers express either optimism or concern (depending on their point of view) of the social effects of Dolto’s advice. Most correspondents praised Dolto for her reassurance and enlightenment that she brought to her audience, like the 63-year old woman from south-western France who wrote of her ‘enormous regret that there was nothing similar to help me avoid several major errors that I committed with my own children’ (September 1977, 7:3:35). But some correspondents expressed objections to pieces of advice that Dolto dispensed, again frequently expressed in terms of trauma. When Dolto made an on-air comment about masturbation in pre-pubescent girls – her position was that there was nothing to be ashamed of, but that one probably shouldn’t do it in public – she attracted a number of outraged letters. ‘Such ideas may be in fashion,’ wrote one woman from the Vendée, ‘but you have serious responsibilities […] masturbation is a grave disorder, which seriously perturbs the potential for future sexual development.’ Another argued that ‘you do not have the right to traumatise people in this way […] what you said was disgusting, scandalous and DANGEROUS’ – and suggested that the girl in question would do better to seek advice from her local priest (April 1977, 5:29). On the other hand, Dolto, a firm believer in the power of the Oedipus complex, was herself accused by other listeners of provoking needless anxiety when she argued that it was ‘dangerous’ for young children to see their parents naked. One wrote that when her children were young she had not made a point of keeping the bathroom door closed, and her children were not obviously traumatised by the experience. Another wrote that ‘there is nothing immoral in nudity […] [but] adults can pervert their children through such taboos of the body’ (July 1977, 6,:40:10-12).

The second type of letter that becomes more noticeable is that in which correspondents discussed their own personal issues at length, seeing Dolto as someone who would understand their problems and perhaps offer support. ‘I think you are the only person who could help me,’ wrote a 17-year old girl suffering with severe depression and alcoholism. ‘Talking to friends does nothing and it is very difficult to ask people for such profound assistance’ (January 1978, 11:20:11). Several such correspondents were not interested or keen on having their case discussed on the radio, but hoped for a written response from Dolto. For some the act of writing was in itself enough: ‘I’m not expecting you to respond to a question because I’m not asking you any, but I had to confide my suffering (*chagrin*) to someone, and my parents and my teachers are too stupid and foolish’ wrote one 18-year old (July 1977, 6:40:9).

These letters frequently manifest a different level of intensity to the rest of the correspondence, and a large proportion of them came from adolescents or young adults. Some speak of the commonplace sufferings of adolescence, such as loneliness, parental incomprehension, yearning for love. ‘I can no longer work at college, I don’t care about the Future, I think only of being Loved, loved truly and profoundly…’ wrote one correspondent (May 1977, 6:35:2). Others raise more profound problems, such as eating disorders, alcoholism and unexpected/unwanted pregnancies. A recurrent theme is the social aspect of distress: these correspondents write to Dolto because they are ill at ease in the world, have no one to confide in, or because their social setting is compounding a previous difficult or traumatic experience. A 15-year-old girl wrote about having been raped the previous year; her current problem, however, was that her parents, shaken by having been unable to protect her, were now no longer willing to let her out of the house. ‘They shout at me and make me work harder than my brothers and sisters. I almost have the impression that they no longer love me’ (April 1978, 13:34:39). One 16-year old described the health problems he had suffered throughout his childhood, as a result of which he now appeared younger and more feminine than his male classmates, who bullied him ceaselessly. ‘For the last seven years my life has been a nightmare […] am I a social misfit [un inadapté], or am I traumatised by the disease that’s blighted me?’ he asked (February 1977, 3:18). A 25-year-old alcoholic from Rennes wrote of his difficult childhood in a rural family and his mother’s early death from lung cancer. But above all, ‘I am gay, a homo [*pédé*] and I think it’s that that’s made me ill and a drunkard […] It’s not easy being a homo in Rennes […] I drink in order to be able to tell guys that I like them and that I want them’ (April 1978, 13:34:92).

* * * * *

Two brief points by way of conclusion. First, although only a small minority of the letters indicate that the writer (or their children) is suffering symptoms of post-traumatic stress, the concept and the language of trauma pervades this correspondence. This shows the extent to which psychological and especially Freudian thinking had entered into popular consciousness: Dolto’s audience understood that neuroses resulted from childhood trauma, and the idea made them anxious about the effects of their parental behaviour. The co-occurrence of the language of trauma with the language of error in the letters, however, suggests that something else may also be at work here, namely Catholic ideas of guilt and original sin. In a rapidly secularising age, Dolto’s correspondents were seeking new sources of guidance and reassurance. In the French context at least, Dolto was a pioneer of using a mass media platform to bring psychoanalytic thinking to a popular audience in search of these qualities (Mehl, 2003).

The second point concerns the power of letter-writing as a form, which performed a cathartic or therapeutic function for at least some writers, in a way that Dolto’s earlier phone-in show could not. Through stimulating the production of thousands of such letters, as much as through any responses that she gave, Dolto helped many people to take a step towards resolving their mental health difficulties. Central to this was Dolto’s status as a trusted interlocutor, someone who would understand. In this sense, Dolto’s position as someone in step with her times and able to bridge the key cultural divide of *les années ‘68*, played an important role in her therapeutic effectiveness.
